# Supplementary material for: Oxidized hemoglobin triggers polyreactivity and autoreactivity of human IgG via transfer of heme
Source: Commun Biol. 2023 Feb 11;6:168. doi: 10.1038/s42003-023-04535-5 (PMC9922299; doi:10.1038/s42003-023-04535-5)
Supplement: Supplementary file 2 — Supplementary Information [file 42003_2023_4535_MOESM2_ESM.pdf]

Supplementary Figure 1.

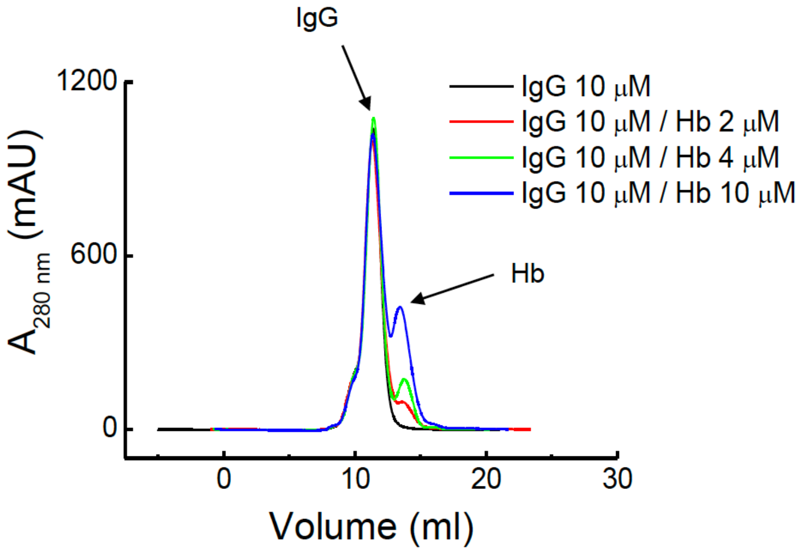

Supplementary Figure 1. Analyses of molecular composition of human pooled IgG after incubation with hemoglobin. Size exclusion elution profiles of native IgG (10 μM) or IgG exposed to 2, 4 and 10 μM of metHb

Supplementary Figure 2.

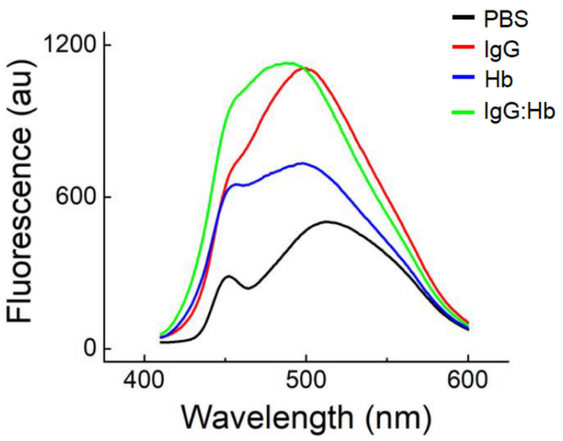

Supplementary Figure 2. Fluorescence spectroscopy analyses of binding of metHb to pooled human IgG. Fluorescence spectra of ANS (10 µM) in buffer only (black line), in presence of 10 µM metHb (blue line), in presence of 10 µM IgG (red line) or in presence of both metHb and IgG (green line).

Supplementary Figure 3.

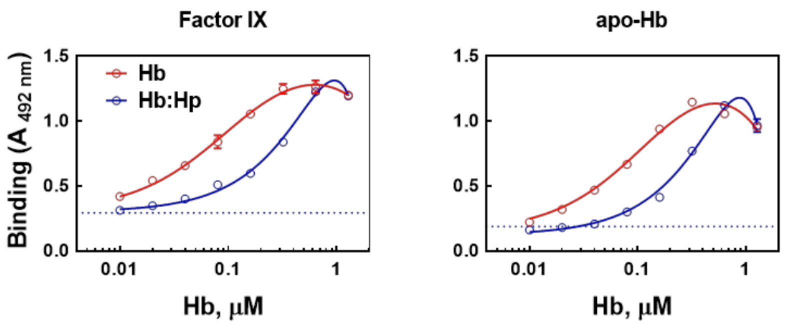

Supplementary Figure 3. Haptoglobin reduces but does not prevent potential of Hb to induce autoreactivity of human IgG. Pooled human IgG at 10 µM weas exposed to increasing concentrations of Hb (0, 0.01 - 1.28 µM) in the absence (red lines and symbols) or in the presence of 0.5 µM of human haptoglobin (blue line and symbols). After incubation for 30 min the samples were diluted 10 × and incubated with surface immobilized factor IX and apo-Hb. Each data point represents mean absorbance ±SD from n=3 repetitions of each sample.

Supplementary Figure 4.

a.

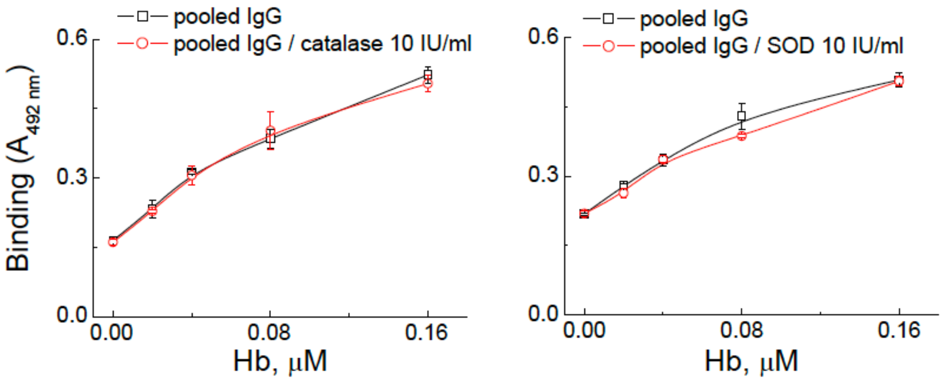

b.

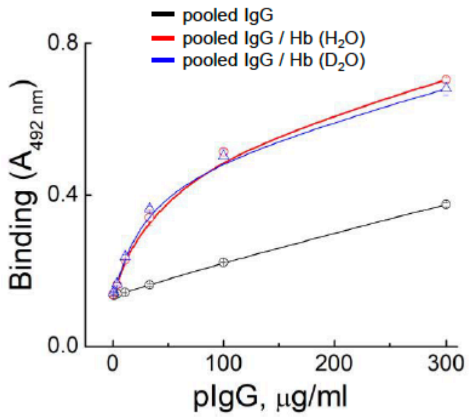

Supplementary Figure 4. Role of pro-oxidative potential of hemoglobin for induction of IgG autoreactivity. Analyses of reactivity of human IgG to immobilized human FIX. **a** Human pooled IgG at 10 µM was exposed to metHb (0 – 0.16 µM) in the presence of 10 IU/ml of catalase or superoxide dismutase. **b** Human IgG at 10 µM was treated with metHb (0.64 µM) in PBS prepared with H_2O or D_2O. Each data point represents mean absorbance ±SD from n=3 repetitions of each sample.

Supplementary Figure 5.

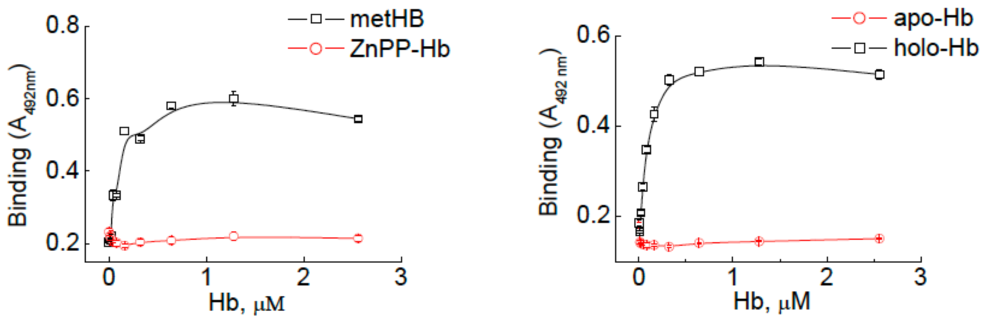

Supplementary Figure 5. Heme is indespensible for modification of antigen-binding specificity of antibodies by Hb. Pooled human IgG) at 10 μM, was exposed to increasing concentrations of metHb, apo-Hb, or apo-Hb reconstituted with Zn(II) protoporphyrin IX. After incubation for 30 min the samples were diluted 10 × and incubated with immobilized factor IX. Each data point represents mean absorbance ±SD from n=4 repetitions of each sample.

Supplementary Figure 6.

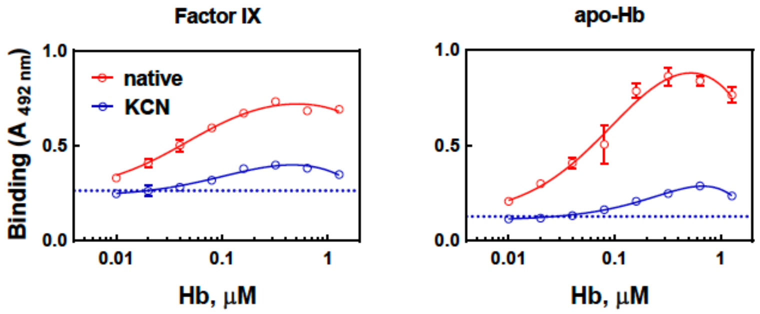

Supplementary Figure 6. Pooled human IgG (left panels) at 10 μM was exposed to increasing concentrations of metHb in the absence (red lines and symbols) or in the presence of 2 mM KCN. After incubation for 30 min the samples were diluted 10 × and incubated with immobilized factor IX and apo-Hb. Each data point represents mean absorbance ±SD from n=3 repetitions of each sample.

Supplementary Figure 7.

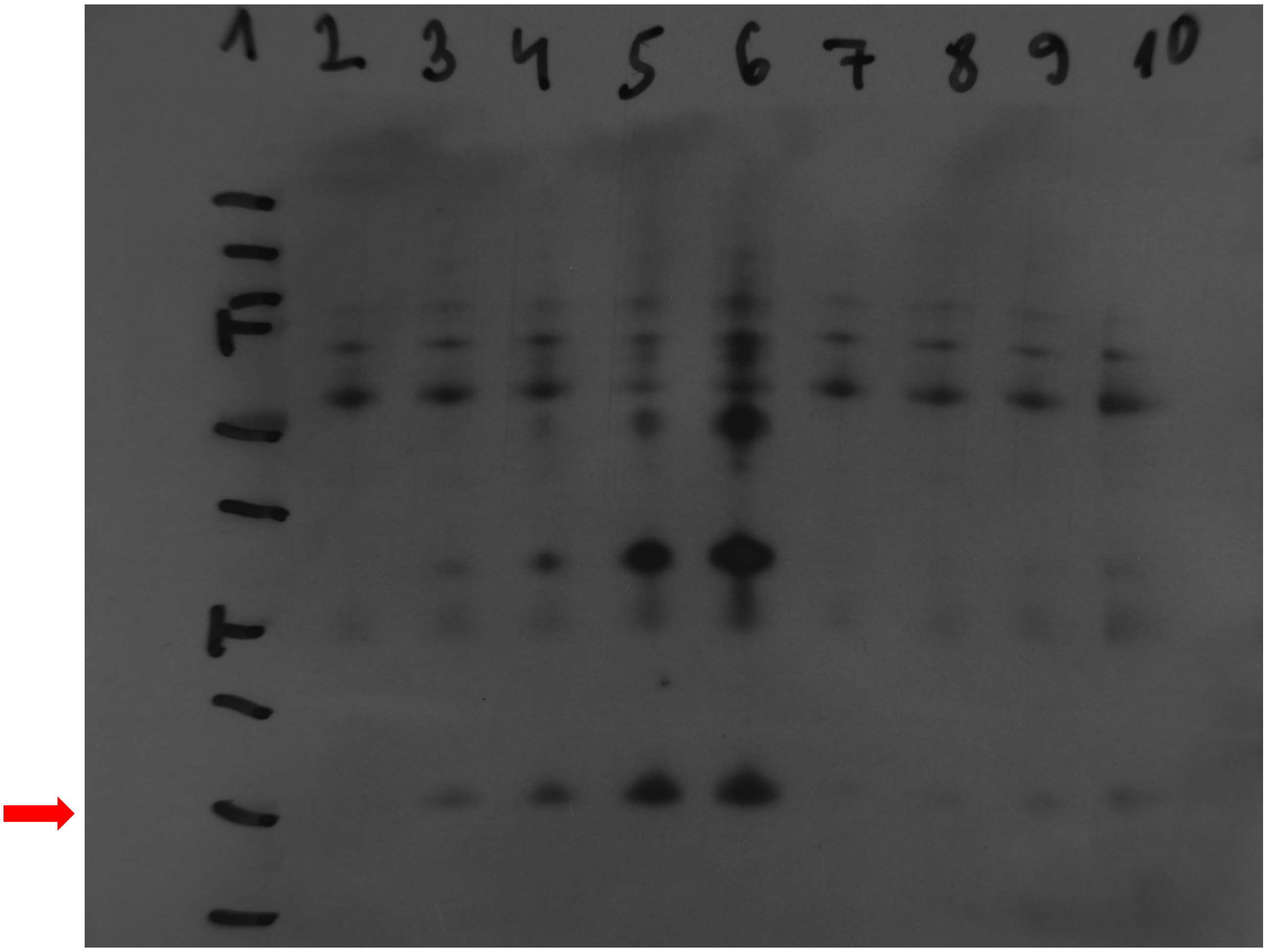

Supplementary Figure 7. Raw data of immunoblot analyses presented on Fig. 1g. Lane 1 depicts MW markers. Red arrow indicates the band of lysosome with a size of 17.5 KDa, with a similar size as the protomers of metHb.
