# Supplementary material for: Oxidized hemoglobin triggers polyreactivity and autoreactivity of human IgG via transfer of heme
Source: Commun Biol. 2023 Feb 11;6:168. doi: 10.1038/s42003-023-04535-5 (PMC9922299; doi:10.1038/s42003-023-04535-5)
Supplement: Supplementary file 3 — Description of Additional Supplementary Files [file 42003_2023_4535_MOESM3_ESM.pdf]

Description of Additional Supplementary Files

File name: Supplementary Data 1

Description: The kinetic values of interaction of heme-exposed Ab21 with metHb and myoglobin

File Name: Supplementary Data 2

Description: Raw data of graphs in main figures and supplementary figures.
